# PCSD1, a new patient-derived model of bone metastatic prostate cancer, is castrate-resistant in the bone-niche

**DOI:** 10.1186/s12967-014-0275-1

**Published:** 2014-10-03

**Authors:** Elana Godebu, Michelle Muldong, Amy Strasner, Christina N Wu, Seung Chol Park, Jason R Woo, Wenxue Ma, Michael A Liss, Takeshi Hirata, Omer Raheem, Nicholas A Cacalano, Anna A Kulidjian, Christina AM Jamieson

**Affiliations:** Department of Urology, University of California, San Diego, La Jolla, CA USA; Department of Surgery, University of California, San Diego, La Jolla, CA USA; Moores Cancer Center, University of California, San Diego, La Jolla, CA USA; Department of Urology, Wonkwang University School of Medicine and Hospital, Iksan, South Korea; Department of Urology, Okayama University Graduate School of Medicine, Dentistry and Pharmaceutical Sciences, Okayama, Japan; Department of Radiation Oncology, University of California, Los Angeles, CA USA; Division of Orthopedic Surgery, University of California, San Diego (UCSD), La Jolla, CA USA

**Keywords:** Xenograft, Bone metastatic prostate cancer, Castrate-resistant, Androgen deprivation therapy, Tumor microenvironment

## Abstract

**Introduction:**

Prostate cancer bone metastasis occurs in 50-90% of men with advanced disease for which there is no cure. Bone metastasis leads to debilitating fractures and severe bone pain. It is associated with therapy resistance and rapid decline. Androgen deprivation therapy (ADT) is standard of care for advanced prostate cancer, however, bone metastatic prostate cancer (PCa) often becomes resistant to ADT. There are few pre-clinical models to understand the interaction between the bone microenvironment and prostate cancer. Here we report the castrate resistant growth in the bone niche of PCSD1, a patient-derived intra-femoral xenograft model of prostate bone metastatic cancer treated with the anti-androgen, bicalutamide.

**Methods:**

PCSD1 bone-niche model was derived from a human prostate cancer femoral metastasis resected during hemiarthroplasty and serially transplanted into *Rag2*^*−/−*^*;*γ_*c*_^*−/−*^ mice intra-femorally (IF) or sub-cutaneously (SC). At 5 weeks post-transplantation mice received bicalutamide or vehicle control for 18 days. Tumor growth of PCSD1 was measured with calipers. PSA expression in PCSD1 xenograft tumors was determined using quantitative RT-PCR and immunohistochemistry. Expression of AR and PSMA, were also determined with qPCR.

**Results:**

PCSD1 xenograft tumor growth capacity was 24 fold greater in the bone (intra-femoral, IF) than in the soft tissue (sub-cutaneous, SC) microenvironment. Treatment with the anti-androgen, bicalutamide, inhibited tumor growth in the sub-cutaneous transplantation site. However, bicalutamide was ineffective in suppressing PCSD1 tumor growth in the bone-niche. Nevertheless, bicalutamide treatment of intra-femoral tumors significantly reduced PSA expression (p < =0.008) and increased AR (p < =0.032) relative to control.

**Conclusions:**

PCSD1 tumors were castrate resistant when growing in the bone-niche compared to soft tissue. Bicalutamide had little effect on reducing tumor burden in the bone yet still decreased tumor PSA expression and increased AR expression, thus, this model closely recapitulated castrate-resistant, human prostate cancer bone metastatic disease. PCSD1 is a new primary prostate cancer bone metastasis-derived xenograft model to study bone metastatic disease and for pre-clinical drug development of novel therapies for inhibiting therapy resistant prostate cancer growth in the bone-niche.

## Background

Prostate cancer is the second leading cause of cancer-related death in men despite advances in prostate cancer management [1]. Since the PSA Era, clinically localized prostate cancer at diagnosis has increased from 73% to 91%, allowing more men to be cured of their organ confined prostate cancer, while metastatic disease at diagnosis has decreased from 6.6% to 4.0% [2]. For the majority of those men who are not cured or who are diagnosed late, however, prostate cancer will metastasize to bone leading to pain, pathologic fractures, spinal compression and rapid decline [3]. Improvements in progression-free survival and cancer-specific mortality rates have been attributed to both earlier detection and improved treatments [2], but the number of men who fail treatment or are diagnosed at later stages is expected to rise again following the USPSTF recommendations and possible decreased utilization of PSA screening.

For recurrent prostate cancer or metastatic disease on presentation, NCCN guidelines recommend starting androgen deprivation therapy (ADT) with close monitoring [4]. Bicalutamide, one form of androgen deprivation, acts as a competitive inhibitor of androgens by binding the androgen receptor (AR), impairing DNA binding to Androgen Response Elements (ARE), and impairing recruitment of co-activators necessary for testosterone or DHT to impart their proliferative effect on responsive cells [5]. At presentation, if metastatic men were started on ADT, 5-year survival increased from 10% to 19% [6]. Unfortunately, prostate cancer bone metastases often become resistant to ADT [7].

Next generation ADTs, chemotherapy, and radiopharmaceuticals, along with supportive care, comprises the next stage of care. There are two FDA-approved next generation ADTs, abiraterone acetate and enzalutamide. Abiraterone inhibits the intra-tumoral androgen production by irreversibly binding the CYP17A1 steroidogenic enzyme in an antagonistic fashion, while enzalutamide binds the androgen receptor with strong affinity and diminishes the nuclear translocation of the androgen receptor. Both have been shown to improve survival [8-11], however, eventually, resistance develops to these as well, perhaps through mutations in the androgen receptor [12]. In fact, in recent clinical trials, the effects of next generation ADT on bone scans were sometimes inconsistent with the favorable biochemical PSA response and patients progressed despite having a biochemical response, that is, a decrease in PSA [13].

There is currently no curative treatment for prostate cancer bone metastases [3]. There are few models to study the mechanisms in which resistance develops within the bone niche and in which to test novel therapies [12]. We have previously described the development and characterization of PCSD1 (Prostate Cancer San Diego 1), a novel patient-derived intra-femoral xenograft model of prostate bone metastatic cancer derived from a surgical specimen from the hip of a patient who was treated with prostate radiation followed by androgen deprivation therapy (ADT) for 2 years in the process of developing castrate resistance [14]. Here, we report an in vivo challenge with ADT in the PCSD1 model and characterize the results to understand further the mechanisms of castration resistance in the bone niche.

## Methods

### Patient-derived xenograft model of bone metastatic prostate cancer

PCSD1 is a patient-derived xenograft model previously described in Raheem *et al*. [14]. Approval was received from the UCSD institutional review board (IRB) to collect surgical bone metastatic prostate cancer specimens for research purposes. The PCSD1 surgical prostate cancer bone metastasis specimen was donated by a man with high risk (Gleason 5 + 5) prostate cancer found to be locally advanced (T3a) and stage 4 (2 of 5 positive lymph nodes, or N1) but without imaging evidence of metastasis underwent prostatectomy with adjuvant ADT and radiation to his prostatic fossa and pelvic lymph nodes. However, approximately 2 years later, he progressed to castrate resistant bone metastatic prostate cancer. He presented with right hip pain and fracture and underwent palliative hemiarthroplasty at which time the surgical prostate cancer bone metastasis was obtained and transplanted into the femurs of 6–8 week old *Rag2*^*−/−*^*;*γ_*c*_^*−/−*^ as described in Raheem et al. [14]. The PCSD1 cells used for experiments herein were freshly isolated from low passage PCSD1 intra-femoral xenograft tumors prepared as described in Raheem *et al.* [14] and re-suspended 1:1 in high concentration Matrigel (BD Biosciences, Inc.) then injected into 6–8 week old *Rag2*^*−/−*^*;*γ_*c*_^*−/−*^ mice either intra-femorally (IF), 50,000 cells per injection of 15 μl into the right femur or one million cells re-suspended 1:1 in high concentration Matrigel per injection of 100 μl sub-cutaneously (SC) in the right flank. All animal protocols were performed under a UCSD animal welfare IACUC approved protocol.

### PCSD1 GLF-lentiviral transduction and in vivo Bioluminescent Imaging (IVIS)

PCSD1 cells were freshly isolated from low passage intra-femoral xenograft tumors and single cell suspensions prepared as previously described [14]. Cells were then transduced with a GFP-luciferase expressing lentiviral vector (GLF), a kind gift from Dr. Catriona Jamieson, UCSD, according to established methods [15,16]. Cells were cultured with virus for 72 hours, sorted by flow cytometry for green fluorescent protein (GFP) positive cells on FACSAria. GFP + cells were resuspended in DMEM/10%fetal bovine serum (FBS) media at 6.7x10^6^ cells/ml and mixed 1:1 in high concentration Matrigel (BD Biosciences, Inc.) and injected into 6–8 week old male *Rag2*^*−/−*^γ_*c*_^*−/−*^ mice either intra-femorally (IF; n = 10) (5 × 10^4^ cells in 15 μl per mouse) or sub-cutaneously (SC; n = 10) (1 × 10^6^ cells in 100 μl per mouse). Mice were monitored weekly for health, body weight and appearance of palpable tumor. Tumor volume was assessed using an in vivo bioluminescence imaging system (IVIS 200; Caliper Inc.) just before euthanizing mice for tissue and tumor collection at the termination of the experiment. Mice were sacrificed at 18 days of treatment or when tumors reached length reached 1.5 cm, maximal allowable size according to UCSD ACP standards.

### Anti-androgen treatment and tumor growth of PCSD1 xenografts

Mice were monitored for health, body weight and appearance of palpable tumor. At 5 weeks after PCSD1 transplantation, the injected mice received an 18-day treatment of daily oral gavage with Bicalutamide (Sigma B9061) 50 mg/kg/day, or vehicle control (0.9% benzyl alcohol (Sigma 402834), 1% Tween-80 (Sigma P4780), 0.5% Methylcellulose (Sigma M0512)). Twice weekly mice were weighed, health status was recorded and the length and width of tumors were measured with calipers. Tumor volume was calculated using the formula:

Tumor volume (mm^3^) = (length (mm) x width (mm)^2^) / 0.52) [17].

### Quantitative RT-PCR

RNA was extracted from flash frozen tissue of xenograft tumors harvested from mice injected IF or SC and treated with Bicalutamide or vehicle. The Qiagen RNeasy kit was used for RNA extraction according to manufacturer recommended protocol [14]. RNA was quantified using NanoDrop. cDNA was synthesized using Superscript III (Invitrogen, by Life Technologies Inc.) and used for quantitative PCR using Light Cycler 480 SYBR-Green I Master kit (Roche Inc). Custom-designed human-specific primers were used for human prostate specific antigen (PSA) and human androgen receptor (AR) were described in Raheem *et al.* [14]. Primers for human prostate specific membrane antigen (PSMA) were 5-GAG GAG CTT TGG AAC ACT GA-3 for the forward primer (PSMA-F1) and 5-CCT CTG CCC ACT CAG TAG AA-3 for the reverse primer (PSMA-R1) (ValueGene Inc.). PSMA, PSA and AR human specific primers were used for PCR amplification of cDNA synthesized with reverse transcriptase (RT+) or without (RT-) and confirmed by DNA sequencing of correctly sized bands. Human and mouse-specific GAPDH or ACTB-specific primers were used as internal reference controls for qPCR [14]. Positive and negative control cell lines included the prostate cancer cell lines, LNCaP, LAPC4, human B cell line, Raji, and 293 T cell lines.

### Immunohistochemistry

Intra-femoral tumors were fixed in 10% formalin, decalcified with 10% EDTA, embedded in OCT and frozen in isopentane/dry ice bath for cryosections or paraffin embedded as described in Raheem *et al.* [14]. Subcutaneous tumors were fresh frozen in OCT or 10% formalin-fixed and embedded in paraffin at the time of harvest. IF and SC sections were H&E and immunostained with anti-PSA, 1:500 (DAKO) [14]. Images were captured using the Aperio ScanScope and Keyence digital microscope. Quantitation of PSA staining in FFPE sections was performed using The Spectrum Analysis algorithm package and ImageScope Analysis software as described in Woo *et al.* [18] with the modifications that five fields of view were randomly selected in Aperio ScanScope digital images at 20 X magnification in each of three different tumors in each treatment group. Total anti-PSA stained area/total analysis area was calculated for each field of view.

### Statistical data analysis

Comparative statistics included non parametric Mann–Whitney U test and Kruskal-Wallis to assess for significance using IBM SPSS Statistics for Windows (Version 21.0.2012 Armonk, NY: IBM Corp). Mouse weights and tumor weights were compared between vehicle and bicalutamide groups. The Mann–Whitney U test was used to compare continuous parameters. Kruskal-Wallis test was used to compare parameters between four groups. The results were considered significant at a p-value of <0.05. All statistical analyses were performed using IBM SPSS Statistics for Windows (Version 21.0.2012 Armonk, NY).

## Results

**Figure 1:**
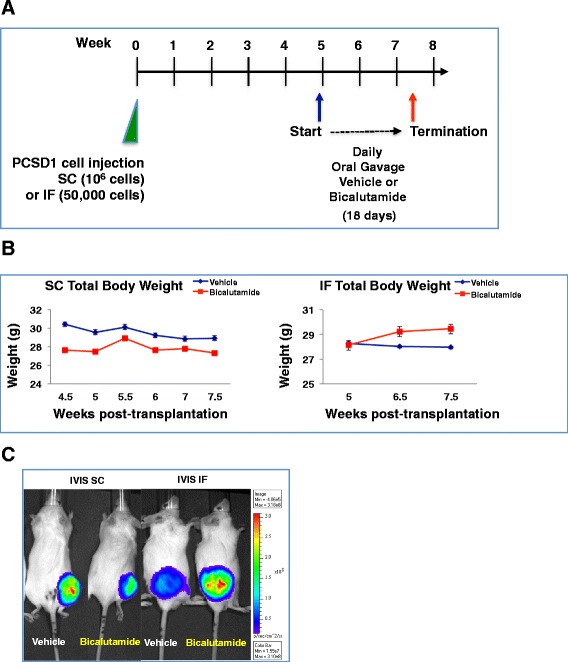
**Testing the anti-androgen response of the PCSD1 prostate cancer xenograft model in bone versus sub-cutaneous niches. A.)** *Rag2* ^*−/−*^ *;*γ_*c*_^*−/−*^ mice were injected with PCSD1 xenograft tumor cells either intra-femorally (IF) or sub-cutaneously (SC) then treated at 5 weeks post-transplantation with bicalutamide or vehicle control for 18 days, **B.)** Changes in total body weights were equivalent in all treatment groups. Mice were weighed biweekly (SC) and weekly (IF); Vehicle control (blue line, SC n = 4 mice, IF n = 5), Bicalutamide 50 mg/kg/day, (red line, SC n = 5, IF n = 5). Differences in mouse weights were not statistically significant (Mann Whitney test, p = 0.095), Error bars denote standard error. **C.)** *In vivo* bioluminescent imaging (IVIS) of PCSD1 tumors which stably expressed GFP-luciferase: SC (left panel) and IF tumors (right panel) at the end of treatment with vehicle and bicalutamide demonstrated preferential castrate-resistance of the PCSD1 tumors in the bone niche. In all the figures, results of vehicle treatment are represented in blue and bicalutamide treatment are represented in red.

The effect of androgen deprivation therapy (ADT) on prostate cancer growth in the bone versus soft tissue microenvironment was tested in the patient-derived xenograft model, PCSD1. Prostate cancer bone metastasis-derived PCSD1 cells were injected directly into the endosteal space of the right femurs (intra-femorally, IF) or under the skin on the right flanks (sub-cutaneously, SC) of 6-8-week old *Rag2*^*−/−*^γ_*c*_^*−/−*^ mice. Treatment with 10 mg/kg/day bicalutamide in a previous experiment had no effect on intra-femoral PCSD1 tumor growth (data not shown). Therefore, a higher dose of bicalutamide (50 mg/kg/day) was tested in these experiments. Treatment with high-dose bicalutamide or vehicle control by daily oral gavage was started when tumors were first palpable at 5 to 5.5 weeks after tumor cell transplantation and continued for 18 days. Mouse total body weights and tumor sizes were measured biweekly. In all the figures, the vehicle treatment is represented as blue and the bicalutamide treatment is represented as red. Total body weights of mice did not change significantly over the treatment period in any of the treatment groups (Figure 1A and B). Differences were not statistically significant when total body weights were compared between vehicle and bicalutamide groups (Final body weight p = 0.095). *In vivo* bioluminescent imaging (IVIS) of tumors was performed in mice just before termination of the experiment and confirmed the presence of PCSD1 tumors (Figure 1C, representative IVIS images).

### PCSD1 Tumor growth in the bone micro-environment was significantly greater than in the sub-cutaneous niche

**Figure 2:**
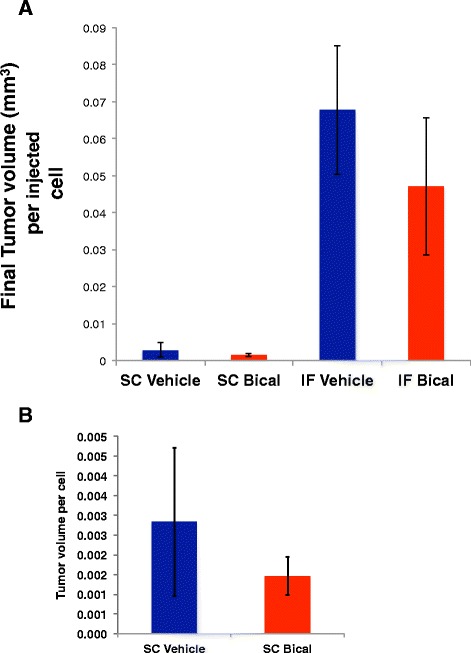
**PCSD1 tumor growth was significantly greater in the bone niche compared to sub-cutaneous niche.** Tumor growth was compared as a function of tumor volume per cell injected at transplantation. The same tumor volumes were attained in the same time when 50,000 PCSD1 cells were injected intra-femorally as when one million PCSD1 cells were injected sub-cutaneously. **A.** Final tumor volume of sub-cutaneous (SC) and intra-femoral (IF) tumors per initially injected cell number. Blue bars show final tumor volume per injected cell in mice treated with vehicle control, Red bars are for mice treated with bicalutamide. There was a 24-fold greater tumor volume per IF-injected cell compared to SC-injected cell in vehicle treated mice and a 16-fold greater tumor volume in bicalutamide treated IF versus SC injected tumors. **B.** Final tumor volume of sub-cutaneous (SC) tumors per initially injected cell number. Error bars show standard deviation.

Tumor growth capacity was compared between the sub-cutaneous versus intra-femoral tumor microenvironments. Tumor volume was measured using calipers biweekly. Strikingly, injection of just 50,000 PCSD1 cells into the femur (IF) resulted in the same sized tumor as the sub-cutaneous (SC) injection of one million PCSD1 cells in the same time period. As shown in Figure 2A, the tumor growth capacity was determined as a function of the number of tumor cells initially injected to attain the same tumor size in the same growth period. There was a 24 fold greater final tumor volume per injected PCSD1 cell in the bone (IF) than in sub-cutaneous (SC) location in mice treated with vehicle control. There was also a 16-fold greater growth of the bicalutamide-treated tumors in the bone-niche than the sub-cutaneous niche. The mean tumor volume per injected cells was greater in the vehicle treated compared to bicalutamide treated mice for both SC and IF transplants, however, this was within standard deviation of the experiments as shown by the error bars. An expanded plot of the sub-cutaneous tumors themselves is shown in Figure 2B. While the means of the tumor volume per injected cell of the bicalutamide treated tumors is lower than the vehicle treated tumors the differences are not statistically significant. Therefore, injection of PCSD1 cells into the bone led to a significantly greater capacity for tumor growth in the bone than in the sub-cutaneous niche.

### Intra-femoral PCSD1 tumor growth was more resistant to treatment with the anti-androgen, bicalutamide

**Figure 3:**
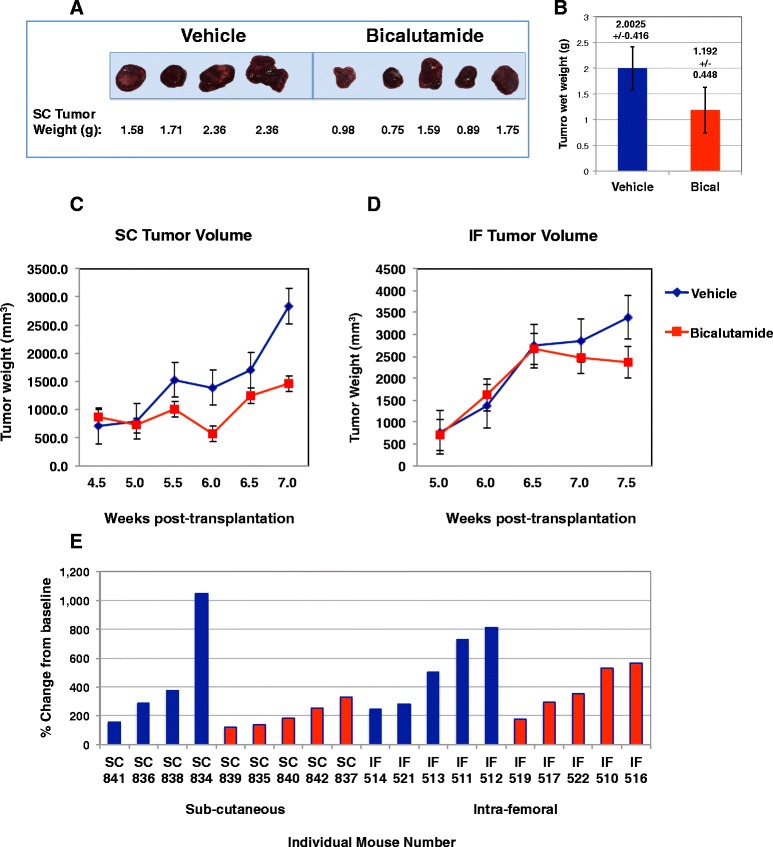
**Bicalutamide treatment inhibited the growth of sub-cutaneous (SC) PCSD1 tumors more than intra-femoral (IF) PCSD1 tumors.** PCSD1 xenograft cells were directly injected either sub-cutaneously (SC) or intra-femorally (IF) into *Rag2* ^*−/−*^ *;*γ_*c*_^*−/−*^ mice. **A.)** Wet tumor weight of bicalutamide treated SC tumors was reduced. **B.)** Bicalutamide reduced average wet SC tumor weight by half. **C.)** Bicalutamide (red line) suppressed PCSD1 tumor growth in mice with sub-cutaneously transplanted tumors compared to vehicle treatment (blue line). **D.)** Bicalutamide (red line) did not significantly suppress PCSD1 tumor growth in mice with intra-femorally transplanted tumors compared to vehicle treatment (blue line). Length and width of tumors were measured in mm using calipers and used to calculate tumor volume. Vehicle control (blue, SC n = 4 mice, IF n = 5), Bicalutamide 50 mg/kg/day, (red, SC n = 5, IF n = 5) **E.)** Waterfall plot of percent change in final tumor volume at termination, Day 18, from baseline, the tumor volume treatment Day 1. Blue bars are for individual mice treated with vehicle control, Red bars are for individual mice treated with bicalutamide. Error bars denote standard error.

Tumor growth over the time of treatment was compared between vehicle and bicalutamide-treated mice. Tumor volume was measured using calipers biweekly. At the termination of the experiment sub-cutaneous (SC) tumors were removed and weighed as shown in Figure 3A. The average tumor weight of bicalutamide-treated SC tumors was almost half of the vehicle-treated SC tumors (Figure 3B). Wet weights of intra-femoral (IF) tumors were not included in this analysis, however, because the femur bone was still encased within the excised tumor mass.

To compare tumor growth rates between groups, biweekly caliper measurements were plotted against time as shown in Figure 3C. The treatment of the SC tumors was started 0.5 weeks earlier in the post-transplantation time course than for the IF tumors. Nevertheless, the total treatment time of 18 days was the same for both SC (4.5 week to 7 weeks post-transplantation) and IF tumors (5 weeks to 7.5 weeks post-transplantation) and the tumor volumes and growth rates for SC and IF tumors at both time points of 4.5 and 5 weeks were equivalent. Bicalutamide treatment led to growth inhibition in SC tumors compared to vehicle treated SC tumors (Figure 3C). The Mann–Whitney statistical analysis indicated that differences in tumor volumes trended towards significance as seen in the p values for each time point in Table 1. In comparison, bicalutamide had almost no effect on the growth rates of IF tumors compared to vehicle (Figure 3D). The percent change in each tumor from Day 1 of treatment (baseline) until termination at 18 days of treatment was greater in the SC tumors than the IF tumors as shown in the waterfall plot in Figure 3E. Therefore, PCSD1 tumor growth in the bone microenvironment was more resistant to bicalutamide than in soft tissue.

**Table 1:** **Statistical analysis of PCSD1 tumor volumes at each time point**

**IF Tumor Volume at Week:**	**5**	**6**	**6.5**	**7**	**7.5**
Vehicle	760.511232	1360.8478	2735.197608	2855.33404	3390.372544
Bicalutamide	699.844704	1614.89276	2663.83624	2463.068608	2358.286112
**p value**	**0.841**	**0.151**	**1**	**0.222**	**0.151**
SC Tumor Volume at Week:	5	5.5	6	6.5	7
Vehicle	791.58599	1529.06611	1390.5021	1693.31617	2833.35351
Bicalutamide	717.73988	1005.140552	568.0168	1243.851128	1460.162808
**p value**	**0.905**	**0.19**	**0.19**	**0.413**	**0.286**

### Bicalutamide reduced expression of Prostate Specific Antigen (PSA) RNA in intra-femoral (IF) PCSD1 tumors

**Figure 4:**
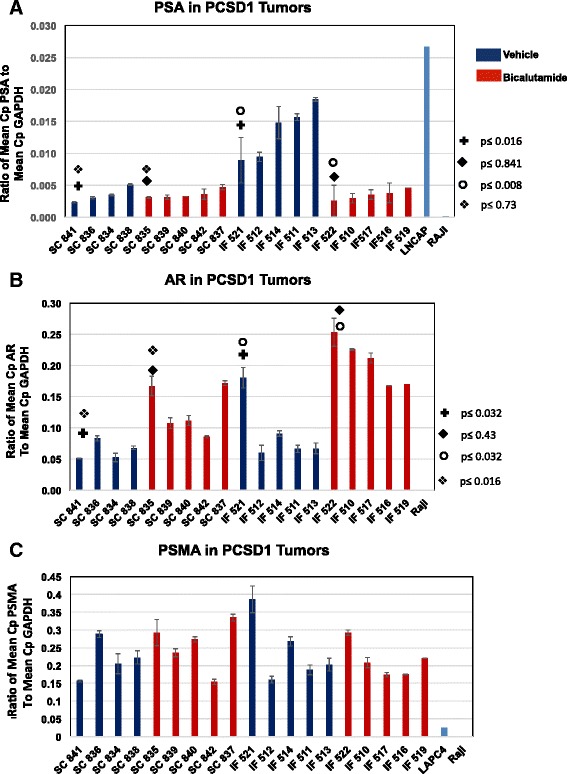
**Bicalutamide reduced expression of prostate specific antigen, PSA, in intra-femoral PCSD1 tumors.** Quantitative RT-PCR was performed on RNA extracted from the intra-femoral and subcutaneous PCSD1 tumors dissected from mice treated with bicalutamide (red bars) or vehicle control (blue bars). Quantitative RT-PCR was normalized to human-specific GAPDH expression levels. The same amount of RNA (1 ug) was used for cDNA synthesis and yield of RNA was similar per PCSD1 and LNCaP cell. The individual mouse numbers are shown on the horizontal axes ranked from low to high PSA level and are in the same order for all three Q-PCR plots, **A-C. A.)** PSA levels were higher in the IF than SC PCSD1 tumor cells. Bicalutamide treatment decreased PSA RNA expression in IF tumors but not SC tumors. Mann Whitney test was used to determine statistical significance (**+**comparison of SC vehicle to IF vehicle treatment p<= 0.016; ◆comparison of SC bicalutamide to IF bicalutamide treatment p<=0.841; ❍ comparison of IF vehicle to IF bicalutamide treatment p<=0.008; ❖ comparison of SC bicalutamide to IF bicalutamide treatment p<= 0.73); **B.)** Bicalutamide increased AR expression in SC and IF tumors (same statistical comparison groups as in **A.)**; **C.)** PSMA expression was unchanged in SC and IF tumors. Human GAPDH was used as an internal reference gene. Ratio of mean Cp of test to mean Cp of GAPDH reference gene is shown. Mann Whitney test was used to determine there was no statistically significant difference between any of the comparison groups. Error bars denote standard error.

The bicalutamide mechanism of action is to inhibit the activity of the androgen receptor (AR) thereby suppressing the viability and proliferation of prostate cancer cells that depend on it. To determine the effect of bicalutamide treatment on AR activity in PCSD1 tumors the expression of AR-target genes was measured using Quantitative RT-PCR (Q-PCR) on RNA extracted from dissected PCSD1 tumors that were transplanted either sub-cutaneously or intra-femorally. Q-PCR was performed on the AR-target gene, prostate specific antigen (PSA, Kallikrein-3), and AR itself. Q-PCR was normalized to human-specific GAPDH expression levels. The same amount of RNA (1 μg) was used for cDNA synthesis and yield of RNA was similar per PCSD1 and LNCaP cell. The individual mouse numbers are shown on the horizontal axes ranked from low to high PSA level and are in the same order for all three Q-PCR plots, Figure 4A-C. As shown in Figure 4A, bicalutamide treatment reduced PSA expression in PCSD1 tumors in the femur even though tumor growth was not significantly reduced (Figure 3D and E). PSA expression in intra-femoral (IF) PCSD1 tumors in vehicle treated mice was comparable to that of the prostate cancer cell line, LNCaP. In contrast, the PSA levels in PCSD1 sub-cutaneous (SC) tumors in both vehicle and bicalutamide-treated mice were significantly lower than in IF PCSD1 tumors and LNCaP. Bicalutamide treatment did not change levels of PSA in SC PCSD1 tumors. Quantitative PCR analysis of AR levels in PCSD1 tumors showed that bicalutamide treatment increased AR expression in both IF and in SC tumors. The level of PSMA, which is highly expressed in many advanced prostate cancers including PCSD1, was unchanged in bicalutamide compared to vehicle treated IF or SC tumors. Therefore, bicalutamide reduced PSA expression in the bone-niche and up-regulated AR expression while tumor growth was largely unaffected.

### Bicalutamide reduced PSA levels in intra-femoral (IF) PCSD1 tumor tissue in immunohistochemical analysis

**Figure 5:**
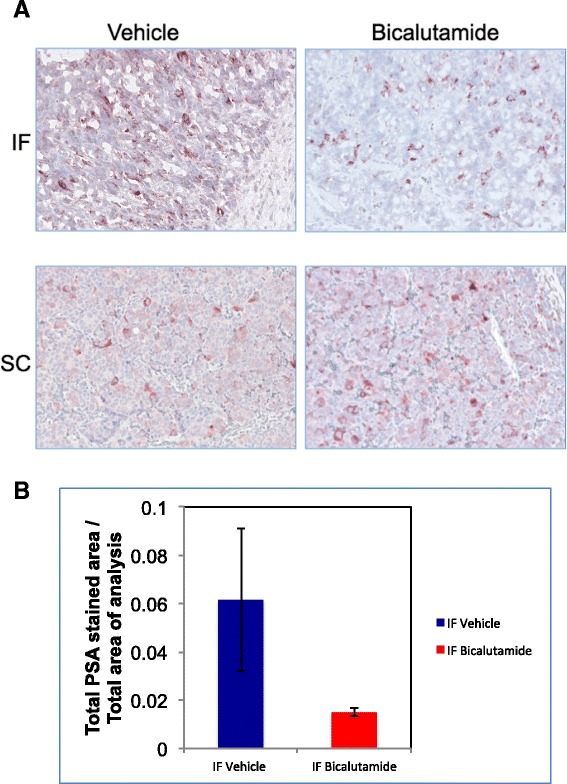
**Bicalutamide-treatment reduced PSA protein expression in intra-femoral PCSD1 tumors in immunohistochemical analysis.** Immunohistochemical analysis of PSA was performed on sections from intra-femoral and sub-cutaneous PCSD1 tumors and counterstained with hematoxylin. Hematoxylin and eosin (H&E) and anti-PSA stains counterstained with hematoxylin were performed on fixed cryosections for intra-femoral tumors and FFPE sections of sub-cutaneous tumors. **A.)** PSA immunostaining intensity was greater in vehicle than in bicalutamide treated intra-femoral (IF) PCSD1 tumors. Magnification was 200×. **B.)** PSA staining was greater in vehicle than bicalutamide- treated PCSD1 intra-femoral tumors. Quantitative digital immunohistochemical analysis of PSA immunostaining intensity was performed and plotted as Total PSA stained area/Total area of analysis. Error bars denote standard deviation.

PCSD1 was derived from a surgical prostate cancer bone metastasis specimen obtained from a man who presented several years before with high risk (Gleason 5 + 5) prostate cancer found to be locally advanced (T3a) and stage 4 (2 of 5 positive lymph nodes, or N1). Pathology on the prostatectomy tissue showed a highly undifferentiated prostate adenocarcinoma with comparatively low PSA levels that was recapitulated in the PCSD1 xenograft tumors [14]. As seen in Figure 5, immunohistochemical analysis of PSA in the dissected PCSD1 tumors showed that bicalutamide treatment decreased the overall PSA immunostaining intensity in the intra-femorally transplanted tumor tissue. Representative images are shown in Figure 5A and demonstrate that PSA levels in the SC tumors were low in both vehicle and bicalutamide treated mice as seen in the qPCR analysis. Semi-quantitative analysis of digitally scanned PSA immunohistochemical staining intensity showed that PSA levels were the same in SC vehicle and SC bicalutamide treated mice whereas PSA levels were reduced in the IF bicalutamide treated compared to IF vehicle treated tumors (Figure 5B). Therefore, changes in PSA protein levels and PSA RNA levels were consistent and demonstrated that bicalutamide treatment reduced PSA in the intra-femoral PCSD1 tumors.

## Discussion

Androgen ablation therapy is standard for advanced prostate cancer, however, bone metastatic prostate cancer (PCa) often becomes resistant to standard-of-care therapies including androgen deprivation, radiation and chemotherapy. Bone metastases of prostate cancers are difficult to biopsy from patients, therefore, the currently available clinical measures of bone metastatic prostate cancer growth in patients are indirect: PSA in the blood and XRays, CT scans which measure bone density or bone scans which measure bone turnover. Changes in bone density and turnover are used as indicators of metastatic tumor growth. There are few pre-clinical models to understand the interaction between the bone microenvironment and prostate cancer. In order to study this directly and to have access to the tumor tissue when it is in the bone we established a new xenograft model of bone metastatic prostate cancer growth, PCSD1, from a surgical prostate cancer bone metastasis specimen (Raheem *et al.* [14]). We tested the anti-androgen, bicalutamide, in PCSD1, our new patient-derived xenograft model of bone metastatic prostate cancer. We transplanted GFP-Luciferase expressing PCSD1 cells intra-femorally (IF) or sub-cutaneously (SC) into immunodeficient mice and provided an androgen deprivation challenge with Bicalutamide. We demonstrated that, as seen in clinical trials of next generation ADT, tumor growth in the bone niche persisted despite a favorable biochemical PSA response. In humans receiving ADT, bone scans have shown persistent growth despite dropping PSA levels [13], and in our mouse model, the Bicalutamide challenge to tumors in the bone-niche did not prevent tumor growth despite the PSA response, while in the subcutaneous tumor, growth slowed. Such a discordance between lowered PSA levels and uncontrolled tumor growth in the bone–niche has also been seen in some abiraterone-treated patients [13].

Thus, this model not only closely recapitulates anti-androgen (castrate) resistant growth of human prostate cancer bone metastasis but also can be used to unravel this paradoxical role of AR in castrate-resistant PCa, which has limited the effectiveness of all androgen deprivation therapies. PCSD1 is a new primary prostate cancer bone metastasis-derived xenograft model to study metastatic disease in the bone and to develop novel therapies for inhibiting androgen deprivation resistant prostate cancer growth in the bone-niche.

In this paper we performed the first direct comparison of the effectiveness of bicalutamide in inhibiting PCSD1 prostate cancer growth in the bone to when it is transplanted elsewhere, in this case, just beneath the skin, or, sub-cutaneously. We found that PCSD1 tumors were more resistant to bicalutamide treatment when growing in the bone compared to sub-cutaneously. Importantly, this is the first report to directly demonstrate a striking (24-fold) greater growth of prostate cancer tumors in the bone-niche than the soft tissue, sub-cutaneous niche.

The implications of these findings are two-fold. First, the finding that the PCSD1 mouse model closely recapitulates the response in humans suggests that this is a good model for studying castrate resistant bone metastatic prostate cancer. Second, the fact that this phenomenon is unique to the bone-niche and is not seen in the same tumor when injected into the subcutaneous tissue confirms previous findings suggesting a unique interaction between prostate cancer and the bone-niche itself. We are currently exploring possible mechanisms for this. While the Mann–Whitney test showed that the differences in tumor growth rates approached statistically significant p values one limitation of this study is that additional animals in the treatment groups should be used.

A small reduction in IF tumor size in the bicalutamide-treated compared to vehicle-treated mice can be seen at the final time point at 18 days of treatment. However, the difference was not statistically significant according to the Mann–Whitney test. More mice are needed to determine whether there is a statistically significant reduction in PCSD1 tumor growth in the intra-femorally transplanted tumors albeit much smaller than the response in the sub-cutaneously transplanted tumors. While longer treatment was not possible due to the tumors having reached the maximal allowed tumor size according to the institutional animal welfare protocol (IACUC), we are currently testing whether earlier treatment may be more effective in suppressing tumor growth in the bone-niche. This may have the clinical implication that bicalutamide treatment in men with any signs of bone metastasis should commence early in the treatment plan. Such a decision would be greatly enhanced with more sensitive, more definitive diagnostic tests for bone metastasis. We are using our PCSD1 model to develop improved imaging of bone metastatic tumors.

The PCSD1 bone-niche xenograft model was derived from a bone metastasis from a patient with castrate resistant disease. PCSD1 is both PSA and AR positive by RNA and protein analysis. Bicalutamide challenge of the PCSD1 bone-niche model led to a decrease in tumor PSA at the level of RNA as well as protein as seen in the immunohistochemistry results. AR compensated by increasing RNA expression. We hypothesize that cross-talk from signaling pathways in the bone microenvironment altered AR activity such that it is still functional even in the presence of androgen deprivation therapies such as bicalutamide. Signaling cross-talk as a mechanism of castrate-resistance in prostate cancer that alters steroid hormone receptor activity and transcriptional regulation of gene expression has been demonstrated for AR in as well as other steroid hormone receptors such as GR [19-23]. Here we show for the first time that the bone microenvironment itself altered AR function and rendered prostate cancer castrate resistant.

An alternative hypothesis is that the bone-niche preference and resulting increased tumor growth is due, at least in part, to the physical enclosure of the cells within the endosteal space in the femur compared to the sub-cutaneous injection where the cells may disperse and, thus, may be readily cleared from the body. This was a concern for the IF injected cells as well since the bone marrow is directly connected to the circulation and cells are known to be rapidly cleared unless they have the correct homing signals. To mitigate such a possibility in these experiments, the SC and IF injected cells were resuspended 1:1 in high concentration Matrigel which rapidly solidifies at temperatures higher than 4°C. Once the cells were injected sub-cutaneously, a solidified mass could be palpated at the injection site which was maintained in most mice for several days to weeks so dispersal of the cells was not immediate, however, it was not necessarily precluded. IVIS imaging is being performed at early time points after initial injection in experiments currently underway to monitor the fate of cells just after injection both sub-cutaneously or intra-femorally. In another study from our lab in which we compared bone lesions in mice injected intra-femorally with Matrigel/Media alone compared to PCSD1 plus Matrigel, immunohistochemical analysis revealed solidified Matrigel still present in the endosteal space of the femurs injected with Matrigel alone at 8 weeks post-injection (Hirata et al., submitted). It is possible that the bone niche enforces and maintains close physical proximity of the tumor cells to each other and to micro-environmental support cells that may be just as important for providing a growth advantage as the signals themselves.

In the near future, many urologists anticipate that there may be a rise in the incidence of advanced prostate cancer since the USPSTF recommendations to stop PSA screening were released. There is no curative treatment for bone metastatic prostate cancer. There are currently few mouse models derived from human castrate resistant bone metastases with serially transplantable mouse xenografts into bone other than PCSD1 which was developed and characterized as previously reported [14]. Here, we confirmed that the castrate-resistant growth of the patient bone metastasis-derived PCSD1 xenograft occurred preferentially in the bone niche.

We investigated the effect of anti-androgen therapy for prostate cancer in the bone versus soft tissue microenvironments using PCSD1, a new patient-derived xenograft model for bone metastatic prostate cancer. We show for the first time that the bone microenvironment itself significantly increased the tumor growth capacity of the prostate cancer cells, altered AR function and rendered the prostate cancer castrate resistant. We demonstrated that, as seen in a sub-set of patients in clinical trials of next generation ADT, tumor growth in the bone niche persisted despite a paradoxically favorable biochemical PSA response.

## Conclusions

PCSD1 is a new patient-derived primary prostate cancer bone metastasis xenograft model from a man who developed castrate resistance metastatic disease. The xenograft is serially transplantable via IF or SC transplantation into Rag2^−/−^;γc^−/−^ male mice, and the xenograft tumors are positive for PSA, PSMA, and AR. We have challenged the xenograft in this model with ADT and recapitulated non-concordant favorable biochemical PSA response despite continued growth in the bone niche. We have demonstrated that this phenomenon is unique to the bone niche, while tumor growth responded favorably to ADT in the subcutaneous setting. Bone microenvironment permits castrate-resistant growth in this xenograft model of bone metastatic prostate cancer. One therapeutic implication of our finding is that prostate cancer therapies need to target potent survival signals that bone provides for prostate cancer, and we are currently working to characterize those signals.
